# A scoping review on the impact of the COVID-19 pandemic on physical activity and sedentary behavior in Saudi Arabia

**DOI:** 10.1186/s12889-023-15422-3

**Published:** 2023-03-27

**Authors:** Kelly R. Evenson, Shaima A. Alothman, Christopher C. Moore, Mariam M. Hamza, Severin Rakic, Reem F. Alsukait, Christopher H. Herbst, Baian A. Baattaiah, Reem AlAhmed, Hazzaa M. Al-Hazzaa, Saleh A. Alqahtani

**Affiliations:** 1 Department of Epidemiology, Gillings School of Global Public Health, University of North Carolina – Chapel Hill, NC Chapel Hill, USA; 2 Lifestyle and Health Research Center, Health Science Research Center, Princess Nourah Bint Abdulrahman University, Riyadh, Saudi Arabia; 3 World Bank Group, Washington, D.C USA; 4 Department of Community Health Sciences, College of Applied Medical Sciences, King Saud University, Riyadh, Saudi Arabia; 5 Department of Physical Therapy, Faculty of Medical Rehabilitation Sciences, King Abdulaziz University, Jeddah, Saudi Arabia; 6 King Faisal Specialist Hospital & Research Center, Riyadh, Saudi Arabia; 7 Division of Gastroenterology and Hepatology, Johns Hopkins University, Baltimore, MD USA

**Keywords:** COVID-19, Physical activity, Middle East region, Saudi Arabia, Sedentary behavior, Surveillance

## Abstract

**Background:**

In Saudi Arabia, stay-at-home orders to address the coronavirus disease 2019 (COVID-19) pandemic between March 15 and 23, 2020 and eased on May 28, 2020. We conducted a scoping review to systematically describe physical activity and sedentary behavior in Saudi Arabia associated with the timing of the lockdown.

**Methods:**

We searched six databases on December 13, 2021 for articles published in English or Arabic from 2018 to the search date. Studies must have reported data from Saudi Arabia for any age and measured physical activity or sedentary behavior.

**Results:**

Overall, 286 records were found; after excluding duplicates, 209 records were screened, and 19 studies were included in the review. Overall, 15 studies were cross-sectional, and 4 studies were prospective cohorts. Three studies included children and adolescents (age: 2–18 years), and 16 studies included adults (age: 15–99 years). Data collection periods were <  = 5 months, with 17 studies collecting data in 2020 only, one study in 2020–2021, and one study in 2021. The median analytic sample size was 363 (interquartile range 262–640). Three studies of children/adolescents collected behaviors online at one time using parental reporting, with one also allowing self-reporting. All three studies found that physical activity was lower during and/or following the lockdown than before the lockdown. Two studies found screen time, television watching, and playing video games were higher during or following the lockdown than before the lockdown. Sixteen adult studies assessed physical activity, with 15 utilizing self-reporting and one using accelerometry. Physical activity, exercise, walking, and park visits were all lower during or following the lockdown than before the lockdown. Six adult studies assessed sedentary behavior using self-report. Sitting time (4 studies) and screen time (2 studies) were higher during or following the lockdown than before the lockdown.

**Conclusions:**

Among children, adolescents, and adults, studies consistently indicated that in the short-term, physical activity decreased and sedentary behavior increased in conjunction with the movement restrictions. Given the widespread impact of the pandemic on other health behaviors, it would be important to continue tracking behaviors post-lockdown and identify subpopulations that may not have returned to their physical activity and sedentary behavior to pre-pandemic levels to focus on intervention efforts.

**Supplementary Information:**

The online version contains supplementary material available at 10.1186/s12889-023-15422-3.

## Background

On March 11, 2020, the World Health Organization declared coronavirus disease 2019 (COVID-19) a pandemic. To tackle viral spread, person-to-person exposure was limited by imposing public movement restrictions. As a result, individuals' physical activity was impacted; there was a drastic change in exercising, walking, and bicycling for transportation and leisure [1]. Sedentary behavior often replaced time spent engaging in physical activity, as the stay-at-home restrictions reduced such opportunities. Even when restrictions were lifted, public facilities (indoor and outdoor) were closed or limited to curtail crowding [2].

The immediate impact of stay-at-home orders on physical activity has been documented through self-reported and device-based metrics. For example, in a convenience sample of 13,503 adults from 14 countries, self-reported moderate-to-vigorous physical activity declined by 41% following pandemic-related restrictions [3]. The decline was greater for work activities than leisure activities, for those with more baseline physical activity compared to those with lesser physical activity, and for younger adults compared to older adults. Other studies using activity trackers indicated immediate declines in step counts (i.e., an indicator of walking levels) attributable to the pandemic in Japan [4], Singapore [5], the United States [6], and worldwide [7–10], although the magnitude of results varied between countries. There is evidence of similar impacts on physical activity among children [1, 11].

The immediate impact of stay-at-home orders has also been documented for sedentary behaviors, characterized as activities while awake with an energy expenditure of 1.5 metabolic equivalents or less while sitting, reclining, or lying [12]. A systematic review found five studies of apparently healthy children and 26 studies of apparently healthy adults, all of which reported increased sedentary behavior primarily due to the pandemic [11]. Another review included 19 studies of children/adolescents and 45 studies of adults and found consistent increases in sedentary behavior during the pandemic period, with larger gains among children compared to adults [13]. The acute global declines in physical activity and increases in sedentary behavior are of concern since engagement in physical activity improves bone health and weight status for children (age 3 to 5 years), improves cognitive function for children/adolescents (age 6 to 13 years), and reduces the risk of mortality, chronic diseases (e.g., certain cancers, cardiovascular disease, obesity), excessive weight gain, fall-related injuries, and dementia for adults [14]. Engagement in sedentary behavior acutely induces vascular dysfunction [15] and, in the long term, increases the risk of mortality, cardiovascular disease, and type 2 diabetes [14, 16].

Physical activity is one of the priority goals of Saudi Arabia’s Vision 2030 given its importance in chronic disease prevention and health benefits [17]. The Saudi Sports for All Federation outlines the vision, framework, goals, and corresponding strategies to help people of all ages become more physically active [18]. The 2021 Household Sports Practice Survey indicated that 48% of the Saudi Arabia population engaged in at least 30 min/week of physical activity, which was higher than the 2019 prevalence of 45% [19]. Two reviews that included studies through early 2018 found that the prevalence of physical inactivity in Saudi Arabia ranged from 55%-96% among children/adolescents, 73%-91% among female adults, and 50%-85% among male adults [20]. A third review included studies published between 2018–2021 that used population-based sampling in the Saudi Arabia population [21]. Among children and adolescents, approximately 80–90% did not attain at least 60 min/day of moderate-to-vigorous physical activity, while for adults approximately 50–95% had a low or insufficient physical activity that did not meet the World Health Organization’s recommendations [22, 23]. In this same review, about 50–80% of children and adolescents engaged in at least two hours/day of screen time or sedentary behavior, while for adults about half had a sitting time of five hours/day or more.

Due to the COVID-19 pandemic, in Saudi Arabia stay-at-home orders were implemented on March 15, 2020, with a suspension of travel for non-essential work, followed by a nationwide curfew from March 23 to April 5, 2020 [24]. The curfews were extended until May 28, 2020, when most regions began easing the curfews. We conducted a scoping review to systematically describe physical activity and sedentary behavior (i.e., physical behaviors) among people of all ages in Saudi Arabia from the pre-COVID-19 pandemic period to the post-movement restriction period. A review focused on Saudi Arabia can bring an understanding of the impact of the pandemic on physical behaviors, identify groups that may not have returned to their pre-pandemic levels, and highlight potential needs for future research and surveillance.

## Methods

### Search methods

The scoping review protocol was developed in accordance with the Preferred Reporting Items for Systematic reviews and Meta-Analyses extension for Scoping Reviews (PRISMA-ScR) statement [25]. Since this review focused on documenting the pandemic-induced change in the prevalence of physical activity and sedentary behavior, and was a scoping rather than a systematic review [26], the protocol was not required to be registered with any platforms. The completed PRISMA-ScR checklist can be found in Supplement 1 [25].

We searched six databases (Cochrane Library, Global Health [EBSCO], PubMed, Scopus, SPORTDiscus [EBSCO], and the World Health Organization Global Index Medicus) on March 3, 2022, with the search strategy detailed in Supplement 2. After manually removing duplicate citations with reference management software, two authors independently screened all titles/abstracts and full-text articles for inclusion using Covidence systematic review software (www.covidence.org; Veritas Health Innovation; Melbourne, Australia), with discrepancies resolved by consensus.

### Inclusion and exclusion criteria

Inclusion criteria were studies that either assessed physical activity or sedentary behavior before and during the pandemic, or asked participants to recall how their physical behaviors changed due to the pandemic. We included studies published between March 1, 2020 and March 3, 2022 that reported on physical activity or sedentary behavior in Saudi Arabia. We included observational studies published in either English or Arabic. Both self-reported and device-based measures of physical activity and sedentary behavior were included in the review.

We excluded studies that did not collect data from March 2020 to March 2022 or did not report the impact of the COVID-19 pandemic on physical behaviors. We also excluded studies that did not report data specifically for Saudi Arabia. We excluded studies that did not include a measure of physical activity or sedentary behavior. For example, studies that discussed “intention to exercise” were excluded since that was not a direct measure of physical behaviors, such as in Alshareef et al. [27]. We excluded studies of hospitalized or institutionalized adults. Grey literature, dissertations, commentaries, and conference proceedings were also excluded.

### Abstraction and analysis

Once the study inclusion was confirmed, one rater abstracted study details and a second rater checked the abstraction, with discrepancies resolved by consensus. The abstraction tool included the study name, study purpose, data collection period, region, sampling methods, target population, inclusion and exclusion criteria, and sample size. Information abstracted on the sample included age, gender, and nationality. We classified age groups based on the predominant age included in the study: children 1 to 12 years, adolescents 13 to 17 years, and adults 18 years and older [28]. For physical activity and sedentary behavior, we collected results at various time points (e.g., before and during lockdown) and the methods used (e.g., questionnaire and definitions).

The quality of each study was assessed to identify strengths and weaknesses. This was performed by having two reviewers answer ten questions about each study, with disagreements between the raters resolved by consensus. We used the Joanna Briggs Institute Prevalence Critical Appraisal Tool Checklist for Prevalence Studies to assess study quality [29], making modifications and additions to fit the purposes of this review (Supplement 3). In recognition that objective quality assessment tools treat each threat to validity equally, [30] we did not intend to provide a total score for each study. Instead, we used the quality assessment results to focus on the specific threats to validity identified across the included studies.

## Results

**Fig. 1:**
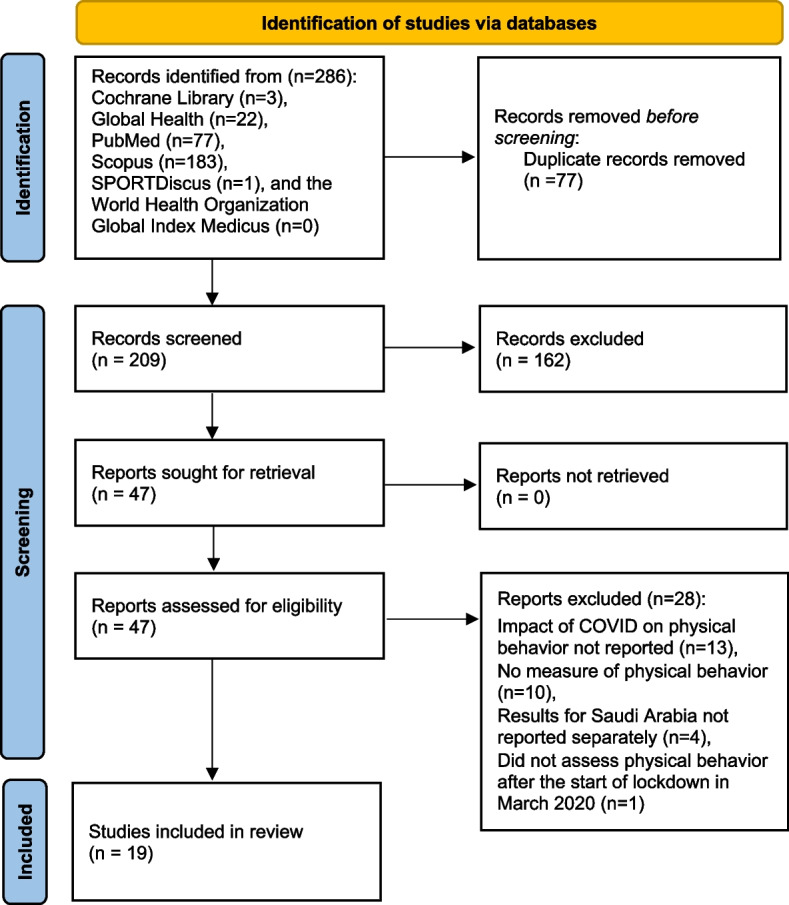
PRISMA flow diagram of the search strategy and results for the scoping review. *From:* Page MJ, McKenzie JE, Bossuyt PM, Boutron I, Hoffmann TC, Mulrow CD, et al. The PRISMA 2020 statement: an updated guideline for reporting systematic reviews. BMJ 2021;372:n71. https://doi.org/10.1136/bmj.n71. For more information, visit: http://www.prisma-statement.org/

A total of 286 records were found; after manually removing 77 duplicates across databases, 209 records were screened for inclusion (Fig. 1). In the title and abstract screening stage, 162 records were excluded as irrelevant. After a full-text review of 47 reports, we included 19 studies [24, 31–48], all published in English.

Most studies were cross-sectional in design, except for four studies that collected data at two [35, 36, 45] or seven [43] time points (Table 1). Data collection mainly occurred in 2020, except for the study by Abdulaziz et al.[32] (data collection: September 2020 to February 2021) and Almugti et al. [38] (data collection: July 2021 to August 2021). Most data collection occurred for three months or fewer, except for Abdulaziz et al. [32] that collected data over five months. Analytic sample sizes ranged from 65 [46] to 30,134 [43], with a median of 363 (interquartile range 262 to 640).

**Table 1:** Description of each study included in the review (*n* = 19)

First Author, Year	Dates of Data Collection	Data Collection Period in Months	Data Collection Time Points	Location in Saudi Arabia	Sampling Procedure	Analytic Sample Size	Age in Years Mean (SD) or Median [IQR]	Age Range in years	Female Percent	Nationality Percent
**Children/Adolescents**										
Almugti, 2021 [38]	July to August 2021	2	1	Jizan 30.0% Riyadh 19.4%, Eastern Province 12.7%, Medina 11.4%, Asir 9.8%, Makkah 8.6%, Bahah 4.1%, Qassim 1.8%, Hail 0.7%, Najran 0.5%, Tabuk 0.5%, Al Jawf 0.3%, Northern Borders 0.2%	Through social media in Saudi Arabia (e.g., Twitter, WhatsApp, and Facebook)	651	9 (4)	3 to 15	41.0%	All parents were Saudi
Hanbazaza, 2021 [44]	June 22 to July 22, 2020	1	1	All different regions across Saudi Arabia	Conducted using online survey distributed via social media (WhatsApp, Twitter, and Snapchat) in Arabic	280	NR	6 to 15	48.9%	NR
Al Agha, 2021 [34]	April 2020 to June 2020	3	1	Jeddah	Patients with type 1 diabetes were contacted via an online virtual pediatric endocrine outpatient clinic	150	12.5 (4.6)	2 to 18	72.0%	NR
**Adults**										
Abd El-Fatah, 2021 [31]	October 2020	1	1	Makkah region 69.7%, Eastern region 20.4%, Riyadh region 9.9%	Mass email via collaborating authors networks, social media engagement (WhatsApp and Twitter), and snowball sampling	363	36.3 (8.5)	20 to 59	65.6%	NR
Abdulaziz, 2021 [32]	September 2020 through February 2021	5	1	Qassim province (cities of Buraidah, Unaizah, and AlRass)	Each city selected 10% of the PHCC in each city using simple random sampling (10P HCCs total; 5 from Buraidah, 3 from Unaizah, and 2 from AlRass). An average of 20 attendees were selected daily at each PHCC and interviewed	299	38.6 (13.1)	18 to missing	72.6%	NR
Abdulsalam, 2021 [33]	NR	NR	1	Jeddah	Online questionnaire was distributed using social media (Facebook, Twitter, Instagram, and WhatsApp) and email communication	472	NR	18 to 59	68.0%	NR
Al Fagih, 2020 [35]	Pre-lockdown (February 2 to March 12, 2020) and lockdown (March 12 to April 19, 2020)	2 pre and 2 post	2	Riyadh	Contacted patients at a cardiac center	82	65 [58 to 72]	NR	35.4%	NR
Alfawaz, 2021 [24]	Two weeks during and after Ramadan (May 11 to June 6, 2020)	1	1	NR, implies all regions	Online questionnaire was cascaded to different social media outlets throughout Saudi Arabia	1965	35.2 (13.1)	15 to 75	53.0%	83.1% Saudi, 16.9% non-Saudi
Al-Musharaf, 2021 [36]	Pre-lockdown (February to April 2019) and lockdown (April to May 2020)	3 pre and 2 post	2	Riyadh	Randomly selected women aged between 19 and 30 years with no history of medical issues from several colleges	297	20.7 (1.4)	19 to 30	100.0%	All Saudi
Alharthi, 2021 [37]	NR	NR	1	All regions	NR; enrolled only Saudis between the ages of 18 and 60	384	NR	18 to 60	50.6%	All Saudi
Alotaibi, 2021 [39]	March 1 to April 30, 2020	2	1	All regions*	Canvassing on social media, local radio stations, and through university mailing lists	22,053	NR	18 to 40	44.5%	NR
Alqurashi, 2021 [40]	March to May 2020	3	1	Region: Eastern 60.1%, Western 23.5%, Central 15.9%, Northern 0.5%	Google forms of the questionnaire were sent to participants to complete via email and social media platforms (Twitter, Telegram, and WhatsApp)	208	NR	18 to 56	88.9%	Saudi 99.5%, Non-Saudi 0.5%
Bakhsh, 2021 [41]	Two week period between June and early July 2020	1	1	Region: Western 70%, Central 16%, Eastern 8%, Southern 5%, Northern 1%	Online questionnaire distributed on various platforms (WhatsApp, Twitter, and email). Questionnaire link was sent to the authors’ relatives, friends, and neighbors to participate in the study and to share the link with their contacts	2,255	NR	18 to missing	64.0%	Saudi 91%, Non-Saudi 9%
Barwais, 2020 [42]	April 9 to April 25, 2020	1	1	Region: Makkah 73.4% and Medinah 26.6%	Convenience sample recruited through email invitations and on social media sites (Twitter, Telegram, and WhatsApp groups)	244	33.8 (7.7)	18 to 50	36.9%	NR
BinDhim, 2021 [43]	Total of 7 waves of collection in 2020	grouped into 4 waves of ~ 3 months each	7 waves; grouped into 4 quarters	All 13 administrative regions	Proportional quota sampling using phone interviews with an age- and gender-stratified random selection of phone numbers from a list generated from the Sharik Association for Health Research (a database of > 80,000 individuals interested in participating in health research, covering all 13 administrative regions)	30,134 over all waves; Quarter 1 = 7050, Quarter 2 = 11,289, Quarter 3 = 5183, Quarter 4 = 6612	36.5 (13.5)	18 to 99	51.2% overall	All Saudi
Jalal, 2021 [45]	Before lockdown (March 2020) and during lockdown (June 2020)	1 pre and 1 post	2	Al-Ahsa	Students of undergraduate programs were selected from their registration numbers by using a simple random technique	628	20.5 (1.9)	18 to 30	70.9%	NR
Magliah, 2021 [46]	Three days after the lockdown ended in Saudi Arabia (June 21–23, 2020)	1	1	Jeddah	Web survey (Google Forms) distributed via social media to patients who were actively attending the specialized insulin pump clinic	65	30 (7.9)	18 to missing	70.8%	All Saudi
Šagát, 2020 [47]	May 10 to May 17, 2020	1	1	Riyadh	Simple randomization to select 1000 potential participants on the Riyadh municipality forum groups that were available on social media who were then sent the online questionnaire	463	35.6 (9.8)	18 to 64	44.1%	71% Saudi citizens and 29% foreigners
Sultan, 2021 [48]	August to September 2020	2	1	NR, implies all regions	Non-probability convenient sample; online survey distributed using social media	338	40 [IQR not reported]	30 to 44	79.0%	NR

*Abbreviations:* *IQR *Interquartile range, *NA* Not applicable, *NR* Not reported, *PHCC* Primary health care clinics, *SD* Standard deviation

^*^obtained directly from authors

Studies were conducted in certain regions of Saudi Arabia such as Al-Ahsa [45], Jeddah [33, 34, 46], Qassim [32], Riyadh [35, 36, 47], or in multiple regions [31, 40–42]. Other studies included participants from the entire country, either stated explicitly [37, 38, 43, 44] or implied [24, 39, 48]. The most common sampling procedure was some combination of convenience sampling through social media platforms, email, radio, or mailing lists [24, 31, 33, 38–42, 44, 46, 48]. Other studies used country-level proportional quota [43] or simple random sampling through health clinics [32, 34, 35], universities [36, 45], or municipal forum groups [47]. The sampling technique was not reported for one study [37].

Three studies included children and adolescents [34, 38, 44], 1 study included participants aged 15 to 75 years (which we assigned to the adult group) [24], and the remaining 15 studies included adults at least 18 years of age [31–33, 35–37, 39–43, 45–48]. All studies enrolled both males and females, except Al-Musharaf et al. [36] who enrolled females only. Some studies enrolled only participants of Saudi nationality [36–38, 43, 46], while others included Saudi and non-Saudi nationalities residing in the country [24, 40, 41, 47]. Ten studies did not report the nationality of participants [31–35, 39, 42, 44, 45, 48].

### Impact on physical behaviors of children/adolescents

Three studies recruited children/adolescents through social media platforms [38, 44] or a pediatric endocrine clinic [34]. The age range was wide for all three studies, from a minimum age of 2 [34], 3 [38], or 6 [44] to a maximum age of 15 [38, 44] or 18 years [34]. All relied on parental reports, although one study also allowed self-reporting [34] (Table 2). The questionnaires were administered online and asked about the daily duration of physical activity [34], daily duration of moderate-to-vigorous physical activity [38], or frequency of participation in physical activity [44] before and during the lockdown. All three studies conducted measurements at a single time point.

**Table 2:** Physical activity results from the scoping review (*n* = 19)

First Author, Year	Physical Activity Assessment Method	Physical Activity Definitions Used	Physical Activity Before Lockdown	Physical Activity During and/or Following Lockdown	Change Reported	Summary
**Children/Adolescents**						
Almugti, 2021 [38]	Parent-reported questionnaire modified by an expert panel	Based on Canadian 24-Hour Movement Guidelines for Children and Youth: At least 1 h/day of MVPA	MVPA < 1 h/day 29%, ≥ 1 h/day 71%	MVPA < 1 h/day 49%, ≥ 1 h/day 51%	P for difference 0.001	Decreased MVPA
Hanbazaza, 2021 [44]	Parent-reported questionnaire	Change in children’s PA (increased, decreased, or remained unchanged); The number of d/wk their children participated in PA before and during lockdown (5 response options ranging from “None at all” to “5–6 times a week”) with PA categorized as ≤ 4 times/week as "not physically active" and ≥ 5 times a week as "physically active"	19.3% were physically active	16.1% were physically active	Proportion of physically active decreased 3.2% but was not statistically significant (*p* = 0.30)	Decreased PA
Al Agha, 2021 [34]	Parent- or self-reported questionnaire	Duration of PA	Daily duration of PA before lockdown: < 30 min 40.5%, 30–60 min 28.0%, Not practicing 27.4%, missing 4.1%*	NR	PA during lockdown: Decreased 66.1%, Increased 19.0%, Not affected 14.9%	Decreased PA
**Adults**						
Abd El-Fatah, 2021 [31]	IPAQ-SF	Changes in PA reported as no change, positive change, or negative change, see footnote below table for definitions	Median PA 380 min/wk; Low 62.3%, Moderate 37.5%, High 0.3%	Median PA 320 min/wk; Low 63.6%, Moderate 34.2%, High 2.2%	Moderate PA days/wk: No change 54.5%, Positive change (increased PA) 25.1%, Negative change (decreased PA) 20.4%; Moderate PA min/day: No change 48.2%, Positive change 32.2%, Negative change 19.6%	Decreased median PA
Abdulaziz, 2021 [32]	Questionnaire	Engagement in any PA, such as walking, going to the gym, and playing sports	NR	58.9% engaging in PA	Change in exercise: Increased 14.0%, Decreased 30.1%, No change 37.8%; Change in park visits: Increased 4.7%, Decreased 76.7%, No change 18.6%	Decreased exercise; Decreased park visits
Abdulsalam, 2021 [33]	Questionnaire translated, tested, and validated by experts at the university	Categorized PA hr/week and PA level per day	Usual daily PA level very low 11.7%, low 17.4%, normal 51.5%, high 17.8%, very high 1.7%	Usual daily PA level very low 22.2%, low 29.0%, normal 34.7%, high 13.1%, very high 0.8%	Usual daily PA level and PA in hr/wk significantly decreased during the COVID-19 period	Decreased usual daily PA level; decreased PA hr/wk
Al Fagih, 2020 [35]	Uniaxial accelerometer embedded in patients' cardiac implantable device (Medtronic ICD/CRT)	PA defined in h/day	Median 2.4 h/day PA	Median 1.8 h/day PA	27.1% decline in PA; change in PA occurred in the first week of March 2020 which coincides with the implementation of social distancing measures	Decreased total PA
Alfawaz, 2021 [24]	Questionnaire designed/revised by multidisciplinary experts and piloted	Categorized daily walking, home physical activities, weight lifting, and swimming: never, 1–2, 3–4, or > 4 days	Daily walking never 21.0%, 1–2 d/wk 23.6%, 3–4 d/wk 24.9%, > 4 d/wk 30.5%; Home PA never 42.8%, 1–2 d/wk 19.0%, 3–4 d/wk 18.0%, > 4 d/wk 20.1%	Daily walking never 23.6%, 1–2 d/wk 22.9%, 3–4 d/wk 24.4%, > 4 d/wk 29.1%; Home PA never 44.6%, 1–2 d/wk 18.8%, 3–4 d/wk 17.1%, > 4 d/wk 19.5%	Significant changes in walking, home physical activities with weights, and swimming (p values < 0.001)	Decreased daily walking; Decreased home activities; Increased swimming
Al-Musharaf, 2021 [36]	GPAQ Arabic version	Meeting recommendation of ≥ 600 MET-min/week	47.5% meeting recommendations	40.7% meeting recommendations	Test for difference in meeting PA recommendations *p* = 0.08	Decreased meeting PA recommendations
Alharthi, 2021 [37]	Modified IPAQ (New Zealand PAQ)	Reported did or did not do exercise	Did exercise 64.2%	Did exercise 48.9%	Increased exercise 48.9% (*p* = 0.01)	Decreased "did exercise"
Alotaibi, 2021 [39]	Questionnaire	Active or inactive based on the WHO guidelines (150–300 min/week of moderate or 75–150 min/week of vigorous intensity PA, or some equivalent combination). MVPA further divided into < 3 d/wk or ≥ 3 d/wk	Inactive 77.1%, Active 22.9%; MVPA > 3 times/wk 13.0%, < 3 times/wk 9.9%	Inactive 80.0%, Active 20.0%; MVPA > 3 times/wk 10.2%, < 3 times/wk 9.8%	Inactive + 2.9%, Active -2.9%; > 3 times/wk -2.9%, < 3 times/wk -0.09%	Decreased active group; increased inactive group
Alqurashi, 2021 [40]	Questionnaire developed and piloted	PA before and during lockdown, asking about: 1) engagement in PA or sports before the pandemic, 2) their gym attendance, 3) whether they exercised at home during the lockdown, 4) whether their time spent exercising increased during the quarantine	Engaged in PA before pandemic 59.1%; exercised at a gym before pandemic 28.8%	Practice exercise at home during lockdown: never 36.5%, 1–3 times/wk 39%, 4–6 times/wk 10.1%, every day 14.4%	During the quarantine period, increased exercise time at home: strongly agree 27.5%, agree 25.5%, neutral 29.8%, disagree 12.5%	Increased time in exercise at home
Bakhsh, 2021 [41]	Questionnaire	Asked participants about changes in their level of PA, weekly frequency of PA, duration of PA per day, and types of PA performed during quarantine	NR	Frequency of PA: none 40%, 1–2 d/wk 22%, 3 d/wk 11%, 4–6 d/wk 14%, daily 13%; Duration of PA: none 40%, < 30 min/d 13%, 30 min/d 15%, 1 h/d 24%, > 1 h/d 8%; Type of PA: walking 65% (most common), cardiorespiratory exercise 11%, and resistance training 7%	Change in PA level: increased 27%, decreased 52%, no change 21%	Decreased PA
Barwais, 2020 [42]	IPAQ-SF	Total MET-min/wk	Mean 903 (SD 755.6) MET-min/wk	Mean 387 (SD 397.8) MET-min/wk	p for paired difference 0.001 with a large effect size (d = 0.89); Social contexts: significant decreases in PA performed alone (*p* < 0.001), with family (*p* < 0.05), with friends (p < 0.05), and with groups (*p* < 0.001)	Decreased PA overall and for men and women
BinDhim, 2021 [43]	Questionnaire refined through linguistic validation, reliability testing, and focus group evaluation	WHO/US PA Guidelines: low level of PA or acceptable PA level (≥ 150 min/wk of MPA and/or ≥ 75 min/wk VPA)	Acceptable PA level Q1 (January to mid-March) 41.0%	Acceptable PA level Q2 (mid-March to June) 26.5%, Q3 (July to September) 24.6%, Q4 (October to December) 24.6%	Significant decline in prevalence odds of having an acceptable PA level between Q1 and each of Q2-4 (all *p* < 0.001) for both unadjusted and adjusted (age, gender, and region) analysis	Decreased PA
Jalal, 2021 [45]	GPAQ	Meeting recommendation of > = 600 total MET-min/wk	Total PA mean 1149.2 (SD 120.08) MET-min/week; Attaining ≥ 600 MET-min/wk 52.1%	Total PA mean 1116.5 (SD 125.3) MET-min/week; Attaining ≥ 600 MET-min/wk 47.9%	differences: MET-min/week *p* = 0.0001, attaining ≥ 600 MET-min/wk *p* = 0.03	Decreased total PA; Decreased meet PA recommendations
Magliah, 2021 [46]	Web survey (Google Forms) with a section asking about the impact of lockdown on different self-management behaviors, which included rating their ability to maintain PA in comparison with the pre-lockdown period	Report change in PA during vs before lockdown in five categories (Greatly decreased to greatly increased)	NR	NR	Greatly decreased 41.5%, somewhat decreased 26.2%, no change 9.2%, somewhat increased 15.4%, greatly increased 7.7%	Decreased PA
Šagát, 2020 [47]	Questionnaire	Categorize their weekly frequency of PA before and after the pandemic: none, once, 2–3 times, 4–5 times, or 6–7 times per week	Did not practice PA 7.3%, PA 1 time/wk 10.3%, PA 2–3 times/wk 35.6%, PA 4–5 times/wk 24.1%, PA 6–7 times/wk 22.7%	Did not practice PA 20.0%, PA 1 time/wk 15.2%, PA 2–3 times/wk 25.1%, PA 4–5 times/wk 25.8%, PA 6–7 times/wk 13.9%	Significant differences in proportions who "did not practice PA" (*p* = 0.001; increase), "practiced PA once a wk" (*p* = 0.02; increase), "practiced PA 2–3 times a wk" (*p* = 0.001; decrease), and "practiced PA 6–7 time a wk" (*p* < 0.001; decrease)	Decreased PA
Sultan, 2021 [48]	Questionnaire developed after a literature review; tested reliability	Categorized participants as not active, light activity, active, or very active (categories not further defined)	Not active 5.3%, light activity 31.2%, active 54.9%, very active 6.8%	Not active 19.0%, light activity 36.2%, active 37.4%, very active 7.4%	"Not active" category increased significantly *p* < 0.001	Increased "not active" category

Abbreviations: *d* days, *ICD/CRT* Implantable cardioverter-defibrillator / cardiac resynchronization therapy, *IPAQ* International PA Questionnaire, *GPAQ* Global PA Questionnaire, *MET* Metabolic equivalent of task, *min* minutes, *MPA* Moderate PA, *MVPA* Moderate to vigorous PA, *NA* Not applicable, *NR* Not reported, *PA* Physical activity, *wk* week, *VPA* Vigorous physical activity, *WHO* World Health Organization

IPAQ-SF November 2005 scoring protocol: Low [not moderate or high]; Moderate [either A) ≥ 3 days of vigorous activity of ≥ 20 min/day, B) ≥ 5 days of moderate-intensity activity or walking of ≥ 30 min/day, or C) ≥ 5 days of any combination of walking, moderate-intensity or vigorous intensity activities achieving ≥ 600 MET-min/week]; High [either A) vigorous-intensity activity on ≥ 3 days achieving ≥ 1500 MET-min/week or B) ≥ 7 days of any combination of walking, moderate-intensity or vigorous intensity activities achieving ≥ 3000 MET-min/week]

^*^obtained directly from authors

The lockdown was associated with a 20-percentage point decrease in moderate-to-vigorous physical activity of at least one hour/day [38] and a three-percentage point decrease in the percent classified as physically active [44]. The third study among participants aged 2 to 18 years with diabetes reported that the lockdown was associated with decreased physical activity for 66.1% of the sample, increased physical activity for 19.0%, and no change for 14.9% [34].

Two studies assessed sedentary behavior by asking parents to report time on digital screens [38] or time spent playing video games and watching television [44] (Table 3). Compared to the pre-lockdown period, the lockdown period was associated with 24.0, 35.0, and 22.5 percentage point increases in the proportion of children/adolescents on screens for > 2 h/day [38], video games for >  = 3 h/day [44], and watching television for >  = 4 h/day [44], respectively.

**Table 3:** Sedentary behavior results from the scoping review (*n* = 8)

First Author, Year	Sedentary Behavior Assessment Method	Sedentary Behavior Definitions Used	Sedentary Behavior Before Lockdown	Sedentary Behavior During and or Following Lockdown	Change Reported	Summary
**Children/Adolescents**						
Almugti, 2021 [38]	Parent-reported questionnaire modified by expert panel	How much time their child spent viewing digital screens, including TV, tablets, and phones; categories based on Canadian 24-Hour Movement Guidelines for Children and Youth: < = 2 h/day of screen time	Use of screens ≤ 2 h/day 37%, > 2 h/day 63%	Use of screens ≤ 2 h/day 13%, > 2 h/day 87%	difference *p* = 0.001	Increased screen time
Hanbazaza, 2021 [44]	Parent-reported questionnaire	How long child played video games and watched TV per day; categorized as > = 3 h/day playing video games (considered high) and > = 4 h/day watching TV (considered high)	Playing video games > = 3 h/day 40.4%; watching TV > = 4 h/day 21.1%	Playing video games > = 3 h/day 75.4%; watching TV > = 4 h/day 43.6%	Significant increases (*p*-values < 0.001) in proportions that were playing video games > = 3 h/day and were watching TV > = 4 h/day	Increased video game time, Increased TV time
**Adults**						
Abd El-Fatah, 2021 [31]	IPAQ-SF	Routine sitting in the day as no change, positive change, or negative change	Daily sitting: 1–2 h/day 20.4%, 3–4 h/day 27%, 5–6 h/day 21.5%, More than 6 h/day 31.1%,	Daily sitting: 1–2 h/day 12.9%, 3–4 h/day 13.8%, 5–6 h/day 18.2%, More than 6 h/day 55.1%,	Daily sitting in h/day: No change 45.5%, Decrease 8.3%, Increase 46.2%	Increased sitting time
Abdulsalam, 2021 [33]	Questionnaire translated, tested, and validated by experts at the university	Time spent in front of the computer, mobile devices, television, etc	< 1 h/day 9.1%, 1–2 h/day 24.2%, 3–4 h/day 33.1%, 5–6 h/day 21.2%, > 6 h/day 12.5%	< 1 h/day 4.7%, 1–2 h/day 7.6%, 3–4 h/day 20.3%, 5–6 h/day 31.1%, > 6 h/day 36.2%	Significant increase (e.g., before 12.5% spent > 6 h/day, but during the pandemic it became the most prevalent category (36.2%)	Increased screen time
Al-Musharaf, 2021 [36]	GPAQ Arabic version	Continuous min/day sitting or reclining	Mean 451.4 (SD 242.1) min/day	Mean 484.9 (SD 257.2) min/day	Test for difference *p* = 0.07	Increase sitting time
Jalal, 2021 [45]	GPAQ	Continuous min/day sitting or reclining	Mean 448.7 (SD 73.6) min/day	Mean 517.8 (SD 83.0) min/day	Differences in min/day *p* = 0.0001	Increased sitting time
Šagát, 2020 [47]	Questionnaire	Asked to categorize their physical behavior at their job/occupation	Occupation: sitting always or most of the time 30.5%, sitting and moving equally 27.9%, moving always or most of the time 42.4%	Occupation: sitting always or most of the time 50.9%, sitting and moving equally 24.2%, moving always or most of the time 24.9%	Significant differences in proportions "sitting always or most of the time" (*p* < 0.001; increase) and "moving always or most of the time" (*p* < 0.001; decrease) during their job/occupation	Increased sitting time at work
Sultan, 2021 [48]	Questionnaire tested for reliability	Daily screen time categorized as < 1 h/day, 1–3 h/day, 4–5 h/day, or ≥ 6 h/day	< 1 h/day 13.4%, 1–3 h/day 48.7%, 4–5 h/day 23.1%, ≥ 6 h/day 14.8%	< 1 h/day 7.1%, 1–3 h/day 29.4%, 4–5 h/day 28.2%, ≥ 6 h/day 35.3%	Increases in proportions with ≥ 6 h/day of screen time and of social media time (p ≤ 0.001 for both)	Increased screen time

Abbreviations: *GPAQ* Global Physical Activity Questionnaire, *IPAQ* *-SF* International Physical Activity Questionnaire short form, *h* hours, *min* minutes, *SD* Standard deviation, *TV* Television

^*^obtained directly from authors

### Impact on physical behaviors of adults

Three studies recruited adults through health clinics [32, 35], and the remaining studies recruited through social media platforms [24, 31, 33, 39–42, 46, 48], universities [36, 45], municipal forum groups [47], proportional quota sampling from throughout the country [43], or was not reported [37]. All but one study relied on self-reported physical activity assessed using questionnaires (Table 2). These included the International Physical Activity Questionnaire [31, 37], the Global Physical Activity Questionnaire [36, 45], or some other questionnaires that experts designed and pilot tested [24, 33, 40, 43], assessed for reliability [48], or not pilot tested (or unreported as such) [32, 39, 41, 46, 47]. The exception was Al Fagih et al. study [35] that enrolled patients with cardiac implantable devices and relied on the accelerometer embedded in those devices for assessing the duration of physical activity for just over a month immediately before and after lockdown. Among 82 patients, median total physical activity declined from pre-lockdown (2.4 h/day) to lockdown (1.8 h/day). The other clinic-oriented study found that 30.1% and 76.7% reported decreases in exercise and park visits, respectively, during the lockdown than before the lockdown [32].

Studies found that fewer adults met recommendations for physical activity [36, 39, 45] or fewer classified themselves as “active or very active” compared to before the lockdown [48]. Studies also found that the lockdown was associated with lower daily or weekly physical activity levels [31, 33, 41–43, 45–47], lower exercise [37], and less daily walking and participation in household activities [24]. Other studies found an increase in swimming [24] and an increase in time spent exercising at home [40] associated with the lockdown. One study explored the results by gender and found that lower physical activity during the lockdown compared to the pre-lockdown period was similar for both women (*n* = 90) and men (n = 154) [42].

Six studies assessed sedentary behavior using a questionnaire (**Table **3). They found that sitting time increased, both overall [31, 33, 36, 45] and while at work [47], and daily screen time increased, all attributable to the lockdown [48]. Specifically, two studies using continuous measures reported an increase in sitting time of 33.5 min/day [36] or 69.1 min/day [45]. Three studies using a categorical measure reported that the proportion of adults with > 6 or ≥ 6 h/day of sitting time [31] or screen time [33, 48] increased by 20.5 to 24.0 percentage points.

### Quality assessment

The quality assessment tool, comprising ten questions and applied to the 19 studies, is provided in Table 4, with the corresponding questions itemized in Supplement 3. All studies had data analysis with sufficient coverage (*n* = 19, question 5), and most measured physical behaviors in a standard way for all participants (*n* = 17, question 8). Most studies described study subjects and the setting in adequate detail (*n* = 14, question 6) and used a valid method to assess the volume of physical behaviors (*n* = 13, question 7). Twelve studies summarized physical behaviors using appropriate analytic methods (question 10), and about half of the studies provided sample size justification (*n* = 10, question 3). However, few studies sampled participants appropriately (*n* = 4, question 2) or provided an appropriate sampling frame to address the target population (*n* = 1, question 1), with most studies recruiting participants through social media platforms, professional networks, health clinics, or universities. Few studies also assessed physical behaviors at least once before and once after lockdown (*n* = 4, question 9) or reported an adequate response rate or appropriately managed non-response (*n* = 3, question 4; 15 did not report on response rate).

**Table 4:** Quality assessment results with studies listed in alphabetical order by the first author's last name (*n* = 19)

**First Author, Year**	**Q1**	**Q2**	**Q3**	**Q4**	**Q5**	**Q6**	**Q7**	**Q8**	**Q9**	**Q10**
**Children/Adolescents**										
Almugti, 2021 [38]	N	N	N	U	Y	Y	Y	Y	N	Y
Hanbazaza, 2021 [44]	N	N	Y	U	Y	Y	N	Y	N	Y
Al Agha, 2021 [34]	N	U	N	U	Y	N	N	N	N	Y
**Adults**										
Abd El-Fatah, 2021 [31]	N	N	Y	U	Y	Y	Y	Y	N	N
Abdulaziz, 2021 [32]	N	Y	Y	Y	Y	Y	U	U	N	U
Abdulsalam, 2021 [33]	N	N	N	U	Y	Y	Y	Y	N	Y
Al Fagih, 2020 [35]	N	Y	N	U	Y	N	Y	Y	Y	Y
Alfawaz, 2021 [24]	N	N	Y	U	Y	Y	Y	Y	N	Y
Al-Musharaf, 2021 [36]	N	Y	N	U	Y	Y	Y	Y	Y	N
Alharthi, 2021 [37]	U	U	Y	U	Y	Y	Y	Y	N	N
Alotaibi, 2021 [39]	N	N	N	U	Y	Y	N	Y	N	Y
Alqurashi, 2021 [40]	N	N	N	U	Y	Y	Y	Y	N	Y
Bakhsh, 2021 [41]	N	N	Y	U	Y	Y	Y	Y	N	Y
Barwais, 2020 [42]	N	N	N	U	Y	Y	Y	Y	N	N
BinDhim, 2021 [43]	Y	Y	Y	N	Y	N	Y	Y	Y	Y
Jalal, 2021 [45]	N	U	Y	U	Y	N	Y	Y	Y	N
Magliah, 2021 [46]	N	N	N	Y	Y	Y	N	Y	N	Y
Šagát, 2020 [47]	N	N	Y	Y	Y	N	N	Y	N	Y
Sultan, 2021 [48]	N	N	Y	U	Y	Y	Y	Y	N	U
Total Yes	1	4	10	3	19	14	13	17	4	12
Total Unclear	1	3	0	15	0	0	1	1	0	2
Total No	17	12	9	1	0	5	5	1	15	5

The quality assessment is available in Supplement 3 of this paper

Abbreviations: *N* No, *U* Unclear, *Y* Yes

## Discussion

This scoping review, based on 19 studies from Saudi Arabia, found consistent evidence across the available literature indicating that physical activity declined and sedentary behavior increased during the COVID-19 lockdown period compared with before. This was most consistent for children, adolescents, and adults across all studies, and was similar for men and women, as reported in one study [42]. For adults, physical activity was lower with the lockdown by approximately 5 to 15 percentage points when considering the studies that classified the proportion as either “active” or “meeting physical activity guidelines” and sedentary behavior was higher by approximately 20 to 25 percentage points for studies that classified the proportion with ~ 6 h/day or more of sitting or screen time.

For children/adolescents, all three studies indicated lower physical activity or moderate-to-vigorous physical activity with the lockdown [34, 38, 44], while two studies indicated a higher proportion of children/adolescents spent time on digital screens [38], playing video games [44], and watching television during the lockdown [44].

There were a couple of notable exceptions among adults, wherein the lockdown was associated with a self-reported increase in swimming [24] and an increase in time spent exercising at home [40]. The higher time spent exercising at home was expected, since time for exercising elsewhere may have been spent at home. Another study reported home exercise among adults but found fewer home activities with the lockdown [24].

We identified four reviews of the global impact of COVID-19 on physical behaviors to compare our results. These reviews found that studies consistently reported lower self-report or device-measured physical activity [1, 11, 49] and higher sedentary behavior [1, 11, 13] associated with lockdown policies. People who were more active prior to the pandemic had larger declines in physical activity [11]. As noted by Stockwell et al.[11], these findings are despite health practitioners and various government organizations guiding how to stay active in self-quarantine and during a lockdown.

The detrimental impact of the lockdown on physical activity was also documented among children/adolescent patients with diabetes [34] and adult patients with heart failure [35] in Saudi Arabia. Other reviews identified studies from different countries that indicated similar declines in physical activity as a result of the lockdown among patients with diabetes, heart failure, congenital heart disease, obesity, and neuromuscular disease [1, 11, 49].

Our review findings are also consistent with an online cross-sectional survey of 2970 adults conducted in April 2020 from the Middle East and North Africa (MENA) region, wherein no physical activity engagement increased from before the pandemic (34.9%) to during the pandemic (39.1%) [50]. Additionally, they found other adverse health impacts over the short term, including weight gain, longer sleep time, and higher reporting of irritability, physical and emotional exhaustion, and tension. Other studies in Saudi Arabia or the MENA region found detrimental impacts of the pandemic on physical health, including changes in eating habits [51], weight gain [51, 52], diabetes [27, 34, 53], and mental health [54–56]. Awareness of increasing physical activity is needed for children, adolescents, and adults in Saudi Arabia [21] since there has been a recent upward trend in obesity and diabetes [57–59]. Taken together with the findings from this review, there is concern over the long-term impact of these observations on physical behaviors. Increasing physical activity and decreasing sedentary behavior is a worthwhile endeavor, given its potential benefit in reducing hospitalizations, admission to an intensive care unit, and death among those with COVID-19 [60, 61].

Physical activity is impacted by the physical environment, including built and natural surroundings [62]. For example, parks provide a crucial place for physical activity and general recreation, particularly in more temperate areas of Saudi Arabia and seasonably cooler times of the year. In our review, one study in the Qassim region found that adults self-reported fewer park visits during the lockdown than before the lockdown [32]. A prospective study in the United States found an increase in park visits at the start of the pandemic, followed by a marked decline in lockdown near closed parks but not near open parks [63]. Once closed parks opened again, their usage increased to levels found at the start of the pandemic. From a worldwide perspective, park visits increased with the start of the pandemic and were lower when the government levied stay-at-home restrictions [2]. If park observation or usage data exist in Saudi Arabia, it would be valuable to investigate whether the patterns followed global trends. Furthermore, an investigation into the impact of the built environment on changing physical behaviors during the pandemic would be worthwhile [64].

### Limitations of the studies included

We found several notable limitations in the current literature that were reviewed, both from the quality assessment (Table 4) and from our observations. First, most studies were cross-sectional, relying on participants to self-report activities before and during the lockdown. Recency bias is a threat to these studies, whereby activities occurring more recently might be easier to report than those occurring more distant in time. Prospective measurement, accomplished in four studies, reduces the risk of bias [35, 36, 43, 45]. This limitation is especially pertinent for two studies that collected data in 2021 [32, 38]. Second, many studies used convenience and nonrepresentative samples, particularly relying on social media platforms for recruitment. While these studies offer the advantage of lower costs and quick access to participants, they also limit participation to those without access to social media platforms and those unwilling to participate using those recruitment channels. This further limits the generalizability of the results.

Third, assessments were mostly based on self-reporting or parental-reporting using a wide variety of questionnaires, precluding our ability to summarize findings with meta-analytic techniques accurately. In fact, many questionnaires appeared non-standardized and did not provide information about the total volume of physical activity. Using valid and reliable metrics for this population would be preferred. Fourth, many studies lacked information on the nationality of their sample, and most studies did not report findings by potential modifiers, such as gender, age, socioeconomic status, region of the country, nationality, or health-associated metrics, possibly due to sample sizes. Fifth, since the period before the COVID-19 pandemic and during the lockdown were not the same seasons of the year, seasonality could confound the relationships observed. Finally, although we included three studies on children and adolescents, the age range was wide and primarily based on parental reporting, which is limited [65]. It would be helpful to document the impacts by narrower age groups to discern any differences that might have a lasting effect on physical behaviors in the future. However, despite these limitations, the findings of the lockdown’s impact on physical behaviors were largely consistent. Future studies are needed to prospectively document physical activity and sedentary behavior changes from pre-pandemic to post-lockdown.

### Strengths and limitations of this review

This scoping review was comprehensive, with searches conducted in six databases, which included Arabic studies, although there were none. To our knowledge, this is the first review to describe the impacts of the COVID-19 pandemic on physical activity and sedentary behavior in Saudi Arabia among children, adolescents, and adults [66]. Based on our inclusion criteria, we accepted all papers regardless of study quality but quantified study quality using a previously developed tool. Despite the strengths of this review, several limitations also exist. The questionnaires used across studies were heterogeneous, as was how they were analyzed, limiting our ability to meta-analyze findings to summarize results. The lack of reporting of findings by sociodemographic and health-related metrics also precluded our ability to summarize across subpopulations.

## Conclusions

In 2021, the Saudi Sports for All Federation set a target to decrease the prevalence of physical inactivity by 30% in adults by 2030 [18]. The findings from this scoping review stress the need to improve physical activity and curtail sedentary behavior in Saudi Arabia, particularly in light of the apparent decline in physical activity and increase in sedentary behavior during and following the COVID-19 lockdown period. This is in agreement with recent worldwide reports on physical activity among children, adolescents, and adults [67–69]. Colleagues have identified the global pattern of unhealthy lifestyle behaviors (including physical behaviors) and the COVID-19 pandemic as a “syndemic”, wherein two or more health conditions or diseases negatively interact [70].

Major areas of focus to support physical activity were designated by the World Health Organization [71] and the International Society for Physical Activity and Health [72]. Some of the Gulf Cooperation Council countries have national policies and strategies to promote physical activity, but implementation is generally low [73]. Others have called for a coordinated regional effort to promote physical activity and reduce sedentary behavior [73]. Consideration should also be given to the specific barriers and facilitators of physical activity and sedentary behavior in Saudi Arabia [74, 75] and the socio-ecologic correlates relevant to this unique time period [76]. Given the widespread impact of the COVID-19 pandemic on other health behaviors, it would be important to continue tracking behaviors and identify subpopulations that may not have returned their physical activity and sedentary behavior to pre-pandemic levels to focus on intervention efforts.

## Supplementary Information

**Additional file 1:** **Table S1.** Preferred Reporting Items for Systematic reviews and Meta-Analyses extension for Scoping Reviews (PRISMA-ScR) Checklist. **Table S2.** Databases searched, search structure, and search terms. **Table S3.** Quality assessment tool applied to each included study.

## Data Availability

All data generated or analyzed during this study are referenced in this published article.

## Abbreviations

COVID-19: Coronavirus disease 2019
MENA: Middle East and North Africa
PRISMA-ScR: Preferred reporting items for systematic reviews and meta-analyses extension for scoping reviews
