# Supplementary material for: A scoping review on the impact of the COVID-19 pandemic on physical activity and sedentary behavior in Saudi Arabia
Source: BMC Public Health. 2023 Mar 27;23:572. doi: 10.1186/s12889-023-15422-3 (PMC10041481; doi:10.1186/s12889-023-15422-3)
Supplement: Supplementary file 1 — Additional file 1: Table S1. Preferred Reporting Items for Systematic reviews and Meta-Analyses extension for Scoping Reviews (PRISMA-ScR) Checklist. Table S2. Databases searched, search structure, and search terms. Table S3. Quality assessment tool applied to each included study. [file 12889_2023_15422_MOESM1_ESM.pdf]

Table S1: Preferred Reporting Items for Systematic reviews and Meta-Analyses extension for Scoping Reviews (PRISMA-ScR) Checklist

SECTION	ITEM	PRISMA-ScR CHECKLIST ITEM	REPORTED ON PAGE #
**TITLE**			
Title	1	Identify the report as a scoping review.	Title, page 1
**ABSTRACT**			
Structured summary	2	Provide a structured summary that includes (as applicable): background, objectives, eligibility criteria, sources of evidence, charting methods, results, and conclusions that relate to the review questions and objectives.	Abstract, page 3-4
**INTRODUCTION**			
Rationale	3	Describe the rationale for the review in the context of what is already known. Explain why the review questions/objectives lend themselves to a scoping review approach.	Introduction, page 5-6
Objectives	4	Provide an explicit statement of the questions and objectives being addressed with reference to their key elements (e.g., population or participants, concepts, and context) or other relevant key elements used to conceptualize the review questions and/or objectives.	Introduction final paragraph, page 6
**METHODS**			
Protocol and registration	5	Indicate whether a review protocol exists; state if and where it can be accessed (e.g., a Web address); and if available, provide registration information, including the registration number.	Methods, paragraph 1 (there is no review protocol), page 6
Eligibility criteria	6	Specify characteristics of the sources of evidence used as eligibility criteria (e.g., years considered, language, and publication status), and provide a rationale.	Methods, page 7
Information sources	7	Describe all information sources in the search (e.g., databases with dates of coverage and contact with authors to identify additional sources), as well as the date the most recent search was executed.	Supplement 2
Search	8	Present the full electronic search strategy for at least 1 database, including any limits used, such that it could be repeated.	Supplement 2
Selection of sources of evidence	9	State the process for selecting sources of evidence (i.e., screening and eligibility) included in the scoping review.	Methods, page 7
Data charting process	10	Describe the methods of charting data from the included sources of evidence (e.g., calibrated forms or forms that have been tested by the team before their use, and whether data charting was done independently or in duplicate) and any processes for obtaining and confirming data from investigators.	Methods, page 7-8
Data items	11	List and define all variables for which data were sought and any assumptions and simplifications made.	Methods, page 7-8
Critical appraisal of individual sources of evidence	12	If done, provide a rationale for conducting a critical appraisal of included sources of evidence; describe the methods used and how this information was used in any data synthesis (if appropriate).	Methods, page 8
Synthesis of results	13	Describe the methods of handling and summarizing the data that were charted.	Methods, page 7-8
**RESULTS**			
Selection of sources of evidence	14	Give numbers of sources of evidence screened, assessed for eligibility, and included in the review, with reasons for exclusions at each stage, ideally using a flow diagram.	Results paragraph 1 page 8, Figure 1
Characteristics of sources of evidence	15	For each source of evidence, present characteristics for which data were charted and provide the citations.	Table 1
Critical appraisal within sources of evidence	16	If done, present data on critical appraisal of included sources of evidence (see item 12).	Table 4

SECTION	ITEM	PRISMA-ScR CHECKLIST ITEM	REPORTED ON PAGE #
Results of individual sources of evidence	17	For each included source of evidence, present the relevant data that were charted that relate to the review questions and objectives.	Table 1-4
Synthesis of results	18	Summarize and/or present the charting results as they relate to the review questions and objectives.	Results section, page 8-11
**DISCUSSION**			
Summary of evidence	19	Summarize the main results (including an overview of concepts, themes, and types of evidence available), link to the review questions and objectives, and consider the relevance to key groups.	Discussion paragraph 1, page 11-12
Limitations	20	Discuss the limitations of the scoping review process.	Discussion, page 14-15
Conclusions	21	Provide a general interpretation of the results with respect to the review questions and objectives, as well as potential implications and/or next steps.	Discussion, page 15
**FUNDING**			
Funding	22	Describe sources of funding for the included sources of evidence, as well as sources of funding for the scoping review. Describe the role of the funders of the scoping review.	Funding section

Abbreviations: JBI, Joanna Briggs Institute; PRISMA-ScR, Preferred Reporting Items for Systematic reviews and Meta-Analyses extension for Scoping Reviews.

Template form from Tricco et al. [25]

Note: page numbers are based on the submitted draft

Table S2: Databases searched, search structure, and search terms

Summary by Source

Source	Date Searched	Number of Results
PubMed	3/3/2022	77
Scopus	3/3/2022	183
SPORTDiscus	3/3/2022	1
Global Health	3/3/2022	22
WHO Global Index Medicus	3/3/2022	0
Cochrane Library	3/3/2022	1 review, 2 trials
**Total (Including Duplicates)**		286
Duplicates Removed		77
**Total (After Duplicates Removed)**		**209**

Search Structure

(Geographical terms connected with *OR*) ***AND*** (physical behavior terms connected with *OR*) ***AND*** (COVID-related terms connected with *OR*)

Filtered for 2020 and later

Table: *Geographical, physical behavior, and COVID-related search terms used*

Geographical terms	Physical behavior terms	COVID-related terms
“Saudi Arabia”	"physical activit*"	"COVID*"
“KSA”	"sedentar*"	"corona*"
“Middle East”	"physical behavior" "physical behaviour"	"nCoV" "2019-nCoV"
“Gulf Cooperation Council”	“inactivity”	"SARS-CoV-2"
“GCC”	“aerobic activit*”	"2019nCoV"
	“endurance activit*”	"HCoV-19"
	“exercise”	"Pandemic*"
	“resistance training”	"outbreak*" "lockdown*"
	“strength training”	
	“walking” “bicycling” “active transport*”	
	“lifestyle”	

Search Terms by Database

Database	Query
PubMed	(“Saudi Arabia”[Title/Abstract] OR “Middle East”[Title/Abstract] OR “Gulf Cooperation Council”[Title/Abstract] OR “GCC”[Title/Abstract] OR “KSA”[Title/Abstract]) AND (“physical activit*”[Title/Abstract] OR “sedentar*”[Title/Abstract] OR “physical behavior”[Title/Abstract] OR “physical behaviour”[Title/Abstract] OR “inactivity”[Title/Abstract] OR “aerobic activit*”[Title/Abstract] OR “endurance activit*”[Title/Abstract] OR “exercise”[Title/Abstract] OR “active transport*”[Title/Abstract] OR “resistance training”[Title/Abstract] OR “strength training”[Title/Abstract] OR “walking”[Title/Abstract] OR “bicycling”[Title/Abstract]) AND (“COVID*”[Title/Abstract] OR “nCoV”[Title/Abstract] OR “2019-nCoV”[Title/Abstract] OR “SARS-CoV-2”[Title/Abstract] OR “2019nCoV”[Title/Abstract] OR “HCoV-19”[Title/Abstract] OR “Coronavirus*”[Title/Abstract] OR “Pandemic*”[Title/Abstract] OR “Outbreak*”[Title/Abstract] OR “Lockdown*”[Title/Abstract] OR “coronavirinae”[Title/Abstract] OR “corona*”[Title/Abstract])
Scopus	TITLE-ABS-KEY(“Saudi Arabia” OR “Middle East” OR “Gulf Cooperation Council” OR “GCC” OR “KSA”) AND TITLE-ABS-KEY(“physical activit*” OR “sedentar*” OR “physical behavior” OR “physical behaviour” OR “inactivity” OR “aerobic activit*” OR “endurance activit*” OR “exercise” OR “active transport*” OR “resistance training” OR “strength training” OR “walking” OR “bicycling”) AND TITLE-ABS-KEY(“COVID*” OR “nCoV” OR “2019-nCoV” OR “SARS-CoV-2” OR “2019nCoV” OR “HCoV-19” OR “Coronavirus*” OR “Pandemic*” OR “Outbreak*” OR “Lockdown*” OR “coronavirinae” OR “corona*”)
SPORTDiscus	(TI "Saudi Arabia" OR AB "Saudi Arabia" OR TI "Middle East" OR AB "Middle East" OR TI "Gulf Cooperation Council" OR AB "Gulf Cooperation Council" OR TI "GCC" OR AB "GCC" OR TI "KSA" OR AB "KSA") AND (TI "physical activit*" OR AB "physical activit*" OR TI "sedentar*" OR AB "sedentar*" OR TI "physical behavior" OR AB "physical behavior" OR TI "physical behaviour" OR AB "physical behaviour" OR TI "inactivity" OR AB "inactivity" OR TI "aerobic activit*" OR AB "aerobic activit*" OR TI "endurance activit*" OR AB "endurance activit*" OR TI "exercise" OR AB "exercise" OR TI "active transport*" OR AB "active transport*" OR TI "resistance training" OR AB "resistance training" OR TI "strength training" OR AB "strength training" OR TI "walking" OR AB "walking" OR TI "bicycling" OR AB "bicycling" OR TI "lifestyle" OR AB "lifestyle") AND (TI "COVID*" OR AB "COVID*" OR TI "corona*" OR AB "corona*" OR TI "nCoV" OR AB "nCoV" OR TI "2019-nCoV" OR AB "2019-nCoV" OR TI "SARS-CoV-2" OR AB "SARS-CoV-2" OR TI "2019nCoV" OR AB "2019nCoV" OR TI "HCoV-19" OR AB "HCoV-19" OR TI "pandemic*" OR AB "pandemic*" OR TI "outbreak*" OR AB "outbreak*" OR TI "lockdown*" OR AB "lockdown*" OR TI "social distancing" OR AB "social distancing")
Global Health	(TI "Saudi Arabia" OR AB "Saudi Arabia" OR TI "Middle East" OR AB "Middle East" OR TI "Gulf Cooperation Council" OR AB "Gulf Cooperation Council" OR TI "GCC" OR AB "GCC" OR TI "KSA" OR AB "KSA") AND (TI "physical activit*" OR AB "physical activit*" OR TI "sedentar*" OR AB "sedentar*" OR TI "physical behavior" OR AB "physical behavior" OR TI "physical behaviour" OR AB "physical behaviour" OR TI "inactivity" OR AB "inactivity" OR TI "aerobic activit*" OR AB "aerobic activit*" OR TI "endurance activit*" OR AB "endurance activit*" OR TI "exercise" OR AB "exercise" OR TI "active transport*" OR AB "active transport*" OR TI "resistance training" OR AB "resistance training" OR TI "strength training" OR AB "strength training" OR TI "walking" OR AB "walking" OR TI "bicycling" OR AB "bicycling" OR TI "lifestyle" OR AB "lifestyle") AND (TI "COVID*" OR AB "COVID*" OR TI "corona*" OR AB "corona*" OR TI "nCoV" OR AB "nCoV" OR TI "2019-nCoV" OR AB "2019-nCoV" OR TI "SARS-CoV-2" OR AB "SARS-CoV-2" OR TI "2019nCoV" OR AB "2019nCoV" OR TI "HCoV-19" OR AB "HCoV-19" OR TI "pandemic*" OR AB "pandemic*" OR TI "outbreak*" OR AB "outbreak*" OR TI "lockdown*" OR AB "lockdown*" OR TI "social distancing" OR AB "social distancing")
WHO Global Index Medicus	(ti:("Saudi Arabia") OR ab:("Saudi Arabia") OR ti:("Middle East") OR ab:("Middle East") OR ti:("Gulf Cooperation Council") OR ab:("Gulf Cooperation Council") OR ti:("GCC") OR ab:("GCC") OR ti:("KSA") OR ab:("KSA")) AND (ti:("physical activit*") OR ab:("physical activit*") OR ti:("sedentar*") OR ab:("sedentar*") OR ti:("physical behavior") OR ab:("physical behavior") OR ti:("physical behaviour") OR ab:("physical behaviour") OR ti:("inactivity") OR ab:("inactivity") OR ti:("aerobic activit*") OR ab:("aerobic activit*") OR ti:("endurance activit*") OR ab:("endurance activit*") OR ti:("exercise") OR ab:("exercise") OR ti:("active transport*") OR ab:("active transport*") OR ti:("resistance training") OR ab:("resistance training") OR ti:("strength training") OR ab:("strength training") OR ti:("walking") OR ab:("walking") OR ti:("bicycling") OR ab:("bicycling") OR ti:("lifestyle") OR ab:("lifestyle")) AND (ti:("COVID*") OR ab:("COVID*") OR ti:("corona*") OR ab:("corona*") OR ti:("nCoV") OR ab:("nCoV") OR ti:("2019-nCoV") OR ab:("2019-nCoV") OR ti:("SARS-CoV-2") OR ab:("SARS-CoV-2") OR ti:("2019nCoV") OR ab:("2019nCoV") OR ti:("HCoV-19") OR ab:("HCoV-19") OR ti:("pandemic*") OR ab:("pandemic*") OR ti:("outbreak*") OR ab:("outbreak*") OR ti:("lockdown*") OR ab:("lockdown*") OR ti:("social distancing") OR ab:("social distancing"))
Cochrane Library	(("Saudi Arabia"):ti,ab OR ("Middle East"):ti,ab OR ("Gulf Cooperation Council"):ti,ab OR ("GCC"):ti,ab OR ("KSA"):ti,ab) AND (("physical activit*"):ti,ab OR ("sedentar*"):ti,ab OR ("physical behavior"):ti,ab OR ("physical behaviour"):ti,ab OR ("inactivity"):ti,ab OR ("aerobic activit*"):ti,ab OR ("endurance activit*"):ti,ab OR ("exercise"):ti,ab OR ("active transport*"):ti,ab OR ("resistance training"):ti,ab OR ("strength training"):ti,ab OR ("walking"):ti,ab OR ("bicycling"):ti,ab OR ("lifestyle"):ti,ab) AND (("COVID*"):ti,ab OR ("corona*"):ti,ab OR ("nCoV"):ti,ab OR ("2019-nCoV"):ti,ab OR ("SARS-CoV-2"):ti,ab OR ("2019nCoV"):ti,ab OR ("HCoV-19"):ti,ab OR ("pandemic*"):ti,ab OR ("outbreak*"):ti,ab OR ("lockdown*"):ti,ab OR ("social distancing"):ti,ab)

Table S3: Quality assessment tool applied to each included study

**Question**
1. Was the sample frame appropriate to address the target population?
2. Were study participants sampled in an appropriate way? (e.g., not convenience sampling)
3. Was the sample size justified and was any estimated sample size requirement achieved?
4. Was the response rate adequate, and if not, was the low response rate managed appropriately?
5. Was the data analysis conducted with sufficient coverage of the identified sample?
6. Were the study participants and the setting described in detail? (Age, gender, some measure of socioeconomic status such as income, occupation, or education)
7. Were valid methods used for the assessment of volume of physical activity/sedentary behavior?
8. Was physical behavior measured in a standard way for all participants?
9. Was physical behavior measured at least once before and once during/after lockdown?
10. Was physical behavior summarized using appropriate analytic methods, including scoring?

All questions were used with or without edits from the Joanna Briggs Institute (JBI) tool detailed in Munn et al. [26]. The exception was question #8 that was added by the authors.

Response options: Yes, No, Unclear
